# Evolution of Plant AIG1-like Proteins: Different Modes of Sequence Divergence and Their Contributions to Functional Diversification

**DOI:** 10.3390/plants15020301

**Published:** 2026-01-19

**Authors:** Jiajing Peng, Liying Xia, Jing Wang, Chunce Guo

**Affiliations:** Jiangxi Provincial Key Laboratory of Improved Variety Breeding and Efficient Utilization of Native Tree Species, Forestry College, Jiangxi Agricultural University, Nanchang 330045, China; ncpengjj@163.com (J.P.); xialiy_2025@163.com (L.X.); wangwang990929@163.com (J.W.)

**Keywords:** *AIG1*-like gene, gene duplication, chloroplast protein import, head-to-head genes

## Abstract

AIG1 (avrRpt2-induced gene 1)-like proteins are a class of GTPases that play crucial roles in plants, functioning both in chloroplast protein import and disease resistance. However, their evolutionary history and the mechanisms driving this functional diversification remain poorly understood. Here, we performed a comprehensive genomic and evolutionary analysis of this gene family across the plant kingdom. We identified 90 *AIG1*-like genes from 11 sequenced plant species, representing major lineages from green algae to angiosperms. Phylogenetic analysis revealed that plant AIG1-like proteins form three monophyletic lineages corresponding to the *Toc34*, *Toc159*, and *IAN* subfamilies, which originated via two ancient duplications predating the divergence of green algae and land plants. These lineages exhibit dramatically divergent evolutionary patterns. The *Toc34* subfamily is evolutionarily conserved, maintaining stable copy numbers and gene structure, indicative of strong functional constraints in its core role in plastid import. In contrast, the *Toc159* and *IAN* subfamilies have undergone dynamic expansion via lineage-specific duplication mechanisms, including segmental duplication and prolific tandem duplication, respectively. Notably, we uncovered a novel mechanism for generating head-to-head tandem duplicates in the *IAN* subfamily, mediated by recombination between inverted repeats. Our analysis of ancestral gene numbers and gene gain/loss dynamics further highlights that functional diversification was driven by both the acquisition of distinct C-terminal targeting domains (M and TM domains) and profound differences in evolutionary rates and duplication modes among subfamilies. This study provides the first full-scale evolutionary framework for plant *AIG1*-like genes, establishing that functional specialization is rooted in distinct modes of sequence and genomic evolution.

## 1. Introduction

Chloroplasts are plant-specific organelles containing the green pigment chlorophyll. They are involved not only in photosynthetic processes such as the photoreduction of carbon, nitrogen, and sulphur, but also in the biosynthesis of various metabolites including fatty acids, amino acids, purine and pyrimidine bases, isoprenoids, and tetrapyrroles [1,2,3]. It is widely accepted that chloroplasts originated through endosymbiosis, involving the incorporation of a single ancestral cyanobacterium into a eukaryotic host (the “Plantae” ancestor) [4,5,6]. The evolutionary success of chloroplasts in Plantae is attributed to two major key innovations: the transfer of a large number of endosymbiont genes to the host nucleus, and the emergence of a chloroplast-specific protein import machinery [7,8]. Typically, modern chloroplast genomes encode only 60–300 genes across different plant lineages, whereas approximately 90% of chloroplast proteins are encoded by nuclear genes [9,10]. The acquisition of this specialized import machinery provided an efficient solution for translocating cytosolically synthesized proteins across the envelope membranes into the chloroplast.

Studies in model plants such as *Pisum sativum* and *Arabidopsis thaliana* have shown that the Translocon at the Outer Chloroplast envelope (Toc) complex is essential for protein import [11,12]. The initial interaction with pre-proteins during the protein import process is regulated by two receptors encoding a unique class of GTPases, namely Toc159 and Toc34 [13,14,15]. Together with the protein-conducting channel Toc75, they constitute the core components of the Toc complex [13]. The Toc75 precursor possesses an N-terminal cleavable bipartite transit peptide that directs it initially to the stroma (though perhaps not completely) and subsequently the outer membrane. Its targeting utilizes the general import pathway prior to insertion into the outer membrane [16,17]. Toc75 is required for the import of nearly all proteins entering the chloroplast.

In *Arabidopsis thaliana*, the *Toc159* family is represented by *atToc159*, *atToc120*, *atToc132*, and *atToc90*, and *Toc34* by *atToc34* and *atToc33* [18,19]. Extensively biochemical and functional studies have demonstrated that different receptor members in *Arabidopsis* tend to mediate the import of distinct types of pre-proteins [19,20,21]. atToc159, the most abundant member Toc159 receptor in chloroplasts, is essential for the quantitative import of photosynthetic proteins. Its knockout mutant *ppi2* displays an albino phenotype and impaired chloroplast biogenesis due to the transcriptional repression of highly expressed photosynthetic genes [21,22,23]. In contrast, the two closely related paralogs, *atToc132* and *atToc120*, play redundant roles in importing non-photosynthetic proteins, and exhibit pre-protein binding properties distinct from those of atToc159 [19,20]. Single mutations in either *atToc132* or *atToc120* do not result in strong visible phenotypes, but double null mutants exhibit structural abnormalities in non-photosynthetic root plastids [19]. Unlike *atToc159*, *atToc132* and *atToc120*, knockout mutants of *atToc90* show no distinguishable phenotype from wild type, despite its uniformly high expression throughout development [19,24]. This suggests that atToc90 protein may play a role unrelated to chloroplast protein import or may have evolved into a pseudogene that lost its protein translocation function.

Interestingly, binding specificity for photosynthetic versus non-photosynthetic proteins has also been observed for the *Arabidopsis* Toc34 receptors. The two *Toc34* genes, *atToc33* and *atToc34*, exhibit functional overlap in the protein import pathway but also display distinct specificities. In vitro assays indicate that atToc33 preferentially facilitates the translocation of photosynthetic precursors and predominates in complexes containing at Toc159, whereas atToc34 is more responsible for non-photosynthetic precursors and is primarily found in complexes containing atToc132/120 [20]. The expression of atToc33 is high in young seedlings but declines rapidly in older plants. In contrast, atToc34 expression remains relatively low throughout plant development [25].

Therefore, investigating the evolution of genes encoding key components of essential protein complexes is a crucial step toward understanding the evolution of the plastid protein import mechanism. The core Toc complex is found in diverse plant species, including *Arabidopsis*, rice, moss, green algae, and red algae, indicating that these proteins are highly conserved and that the protein import machinery evolved prior to the divergence of red algae and green plants [12,26,27,28]. Both Toc33 and Toc159 contain an AIG1 domain and belong to the *AIG1* gene family [29,30]. Previous studies suggest a eukaryotic host origin for these proteins [27]. AvrRpt2-Induced Gene 1 (AIG1)-like proteins are a class of guanosine triphosphatases (GTPases) that function as molecular switches, activated by GTP and inactivated by hydrolysis of GTP to guanosine diphosphate (GDP). These proteins are known to be sequence-conserved. All AIG1-like proteins are characterized by a common AIG1 domain, which consists of five GTP-binding motifs (G1-G5) and a conserved hydrophobic box between G3 and G4 that is unique to this protein family [31,32].

Unexpectedly, another group of plant proteins also harbors the AIG1 domain and functions in resistance responses. These are also known as GTPases of Immunity-Associated Proteins (GIMAPs) or Immune-Associated Nucleotide-binding proteins (IANs). For example, *Arabidopsis thaliana AIG1* is expressed during infection by avirulent strains of *Pseudomonas* that trigger defense responses, including programmed cell death in the infected area, suggesting its involvement in regulating cell death following plant self-defense responses against bacterial infection [32,33]. Immunity-associated *AIG1*-like genes have also been identified in other plants such as maize (*Zea mays*), soybean (*Glycine max*), and tobacco (*Nicotiana tabacum*) [31,34].

Thus, the AIG1 domain defines a protein family with two clearly divergent functional branches: chloroplast protein import and plant immunity. However, the evolutionary relationship between the Toc-associated AIG1 proteins (Toc159 and Toc34) and the immunity-related AIG1 proteins (IANs) remains poorly understood. Previous phylogenetic studies have been largely limited to angiosperms [32], leaving several key questions unresolved: (1) What is the origin and duplication history of the three major AIG1 subfamilies? (2) Why do they exhibit dramatically different evolutionary patterns, ranging from strong conservation to rapid lineage-specific expansion? (3) What gene duplication mechanisms (such as tandem, segmental, or retroposition) underlie their diversification? The broad availability of whole-genome sequences across diverse plant lineages now offers an excellent opportunity to address these questions. In this study, we conducted an extensive identification of AIG1-like proteins from 11 plant model species and performed a detailed phylogenetic analysis. Based on our results, we inferred the origin and evolutionary history of AIG1-like proteins in plants, estimated the number of ancestral genes for each subfamily, and identified evolutionarily conserved and divergent gene lineages. We determined the types of gene duplications that occurred and proposed a mechanism for the generation of head-to-head tandem duplicated genes. Furthermore, we discuss the potential functional implications of the evolutionary patterns observed in this protein family.

## 2. Results

### 2.1. Abundance and Domain Architecture of AIG1 Domain-Containing Proteins

To investigate the evolution of plant *Toc34*, *Toc159*, and *IAN* genes, we retrieved a total of 88 AIG1 domain-containing sequences from 10 sampled plant species. These species represent nearly all major lineages within the plant kingdom, spanning a long evolutionary timeframe from relatively primitive green algae, mosses, and lycophytes to highly derived angiosperms. Our HMMsearch results indicate that species at the base of the plant kingdom possess fewer *AIG1* genes compared to land plants. For instance, only two AIG1 domain-containing proteins were identified in the green alga *Ostreococcus lucimarinus*, and three in *Micromonas pusilla*. In contrast, land plants harbor a relatively higher number of these proteins, albeit with considerable variation in copy number. We identified seven AIG1 domain-containing genes in the moss *Physcomitrella patens*, but only four in the lycophyte *Selaginella moellendorffii*. Among angiosperms, the monocots *Oryza sativa* and *Sorghum bicolor*, as well as the eudicot *Vitis vinifera*, possess 6–8 *AIG1* genes, which is similar to that in *P. patens*. However, copy numbers are significantly higher (2–3 times greater than in rice, sorghum, and grape) in three other eudicot species: *Populus trichocarpa* (12 genes), *Arabidopsis thaliana* (19 genes), and *A. lyrata* (20 genes). Furthermore, AIG1 domain-containing proteins were also identified in animals and protists. We found seven in humans, and one and two in the protists *Tetrahymena thermophila* and *Trichomonas vaginalis*, respectively. These findings demonstrate that AIG1 domain-containing genes are widespread across diverse eukaryotic organisms and exhibit substantial copy number variation. Land plants and higher animals generally possess more *AIG1* genes than other eukaryotes. Given the early divergence of plants and animals within eukaryotes, and the fact that primitive plant representatives (green algae) possess very few copies, it is likely that the increase in *AIG1* gene copy number occurred independently during the evolution of land plants and higher animals.

**Figure 1:**
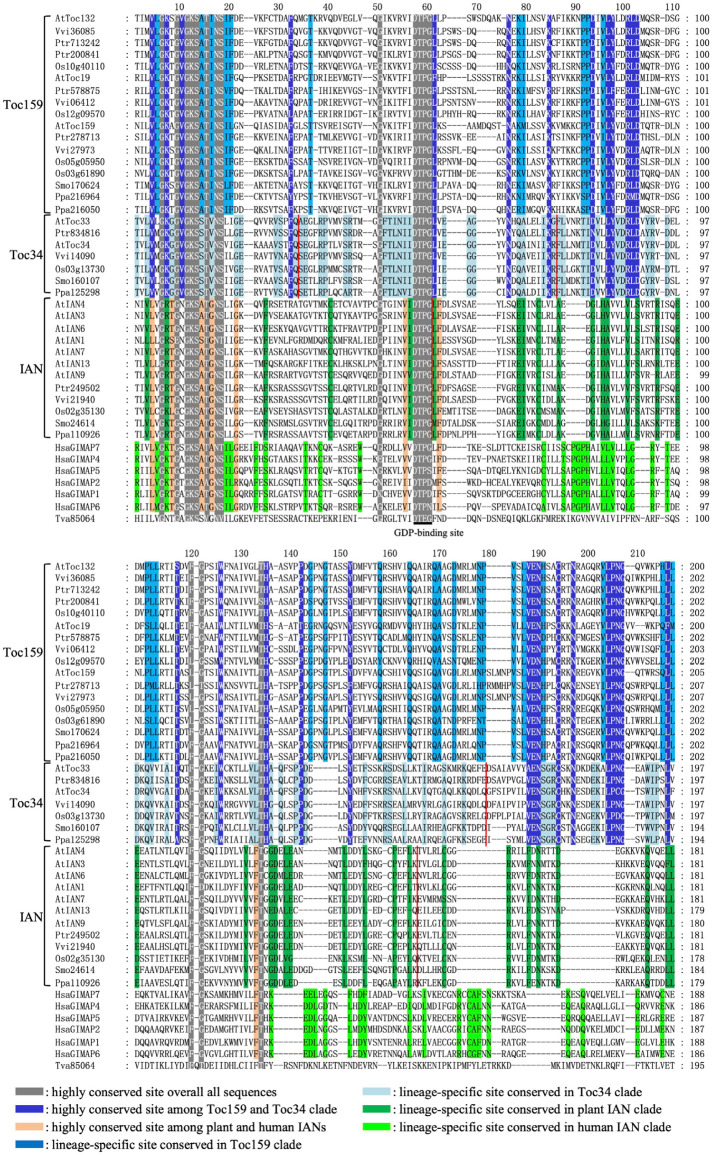
Multiple-sequence alignment of the AIG1 domain. Representative sequences from eight species are shown. The red vertical line indicates an exon boundary. Conserved residues are highlighted with shading to denote varying degrees of conservation.

To characterize the conservation and divergence among Toc34, Toc159, and IAN homologous proteins, we performed a multiple-sequence alignment of all retrieved amino acid sequences (Figure 1). The alignment revealed that the AIG1 domain is highly conserved overall, with a total of 16 nearly invariant amino acid sites. Notably, the residues D57, T58, P59, and G60, which are predicted to form the GDP-binding site, are absolutely conserved across all protein sequences from protists, animals, and plants (Figure 1). Based on the alignment of the AIG1 domain region, plant AIG1 domain-containing proteins can be classified into three distinct lineages, designated IAN, Toc34, and Toc159. Proteins in the IAN lineage contain 35 lineage-specific conserved amino acid sites and show higher similarity in this region to human AIG1-like proteins, sharing eight conserved amino acid sites. Proteins of the Toc34 and Toc159 lineages are more similar to each other within the AIG1 domain than to IAN proteins and share 28 conserved sites that distinguish them from the IAN lineage. Additionally, Toc159 and Toc34 proteins possess 29 and 65 lineage-specific residues, respectively.

Previous studies indicate that the C-terminal M domain of Toc34 and the TM domain of Toc159 are responsible for subcellular localization within the plastid protein import pathway. Using SMART, Pfam, and MEME analyses, we confirmed that the M domain is specific to the Toc34 lineage. This domain is predominantly composed of hydrophilic amino acid residues and functions as a membrane anchor (Figure S1A). Conversely, the TM domain is specific to the Toc159 lineage; it spans 241–244 mainly hydrophobic amino acids and serves as a transmembrane targeting signal (Figure S1B). Furthermore, we identified 18 invariable amino acid sites within the M domain of Toc34 proteins and 51 within the TM domain of Toc159 proteins. This strong conservation strongly suggests that these domains are functionally important across all green plants. Since our analysis indicates that IAN proteins contain only an AIG1 domain and share more sites with human AIG1 domain-containing proteins, we speculate that *IAN* genes may represent the ancestral state of plant *AIG1* genes.

### 2.2. Phylogenetic Relationships of Plant Toc34, Toc159 and IAN Genes

**Figure 2:**
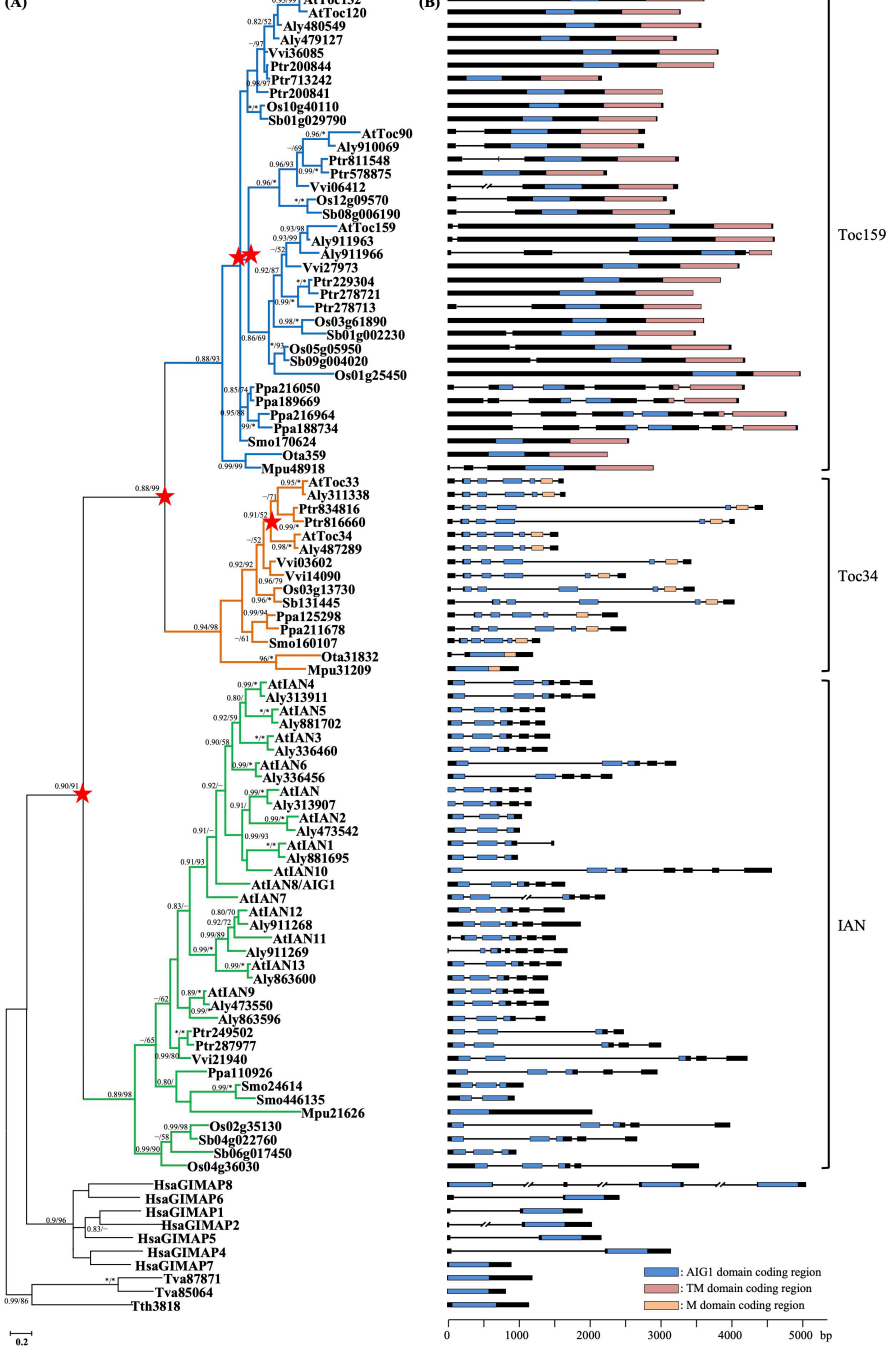
Phylogenetic relationships and gene structure of *AIG1*-like genes. (**A**) Phylogenetic tree of 90 AIG1 proteins from 11 representative species, based on the AIG1 domain region. The topology shown was inferred using Bayesian Inference (BI). Branch support is indicated as follows: asterisks (*) correspond to nodes with a posterior probability (PP) of 1.00 in BI analysis or 100% bootstrap support (BS) in Maximum Likelihood (ML) analysis; dashed lines (–) indicate nodes with PP < 0.50, BS < 50%, or topological conflict between the BI and ML trees. The scale bar represents the number of substitutions per site. The red star represents ancient gene duplication event. (**B**) Exon–intron structure of genes corresponding to the phylogeny in (**A**). Exons are shown as boxes and introns as connecting lines. The coding regions for the AIG1, TM, and M domains are highlighted in different colors.

To elucidate the evolutionary relationships among the *IAN*, *Toc34*, and *Toc159* lineages, we conducted phylogenetic analyses based on an alignment of the AIG1 domain from all 98 identified sequences across 13 selected species. In this study, neighbor-joining (NJ), maximum likelihood (ML), and Bayesian inference (BI) methods yielded consistent results. AIG1 domain-containing genes from plants, humans, and protists each formed monophyletic groups with strong statistical support (high bootstrap values in NJ/ML trees and high posterior probabilities in the BI tree). Furthermore, plant AIG1 domain-containing genes were resolved into three distinct lineages—*IAN*, *Toc34*, and *Toc159*—corroborating the classification suggested by the protein sequence analysis (Figure 2). Each lineage contains representatives from angiosperms, lycophytes, mosses, and green algae. This pattern indicates that the *Toc34* and *Toc159* genes (involved in the plastid protein import pathway) and the *IAN* genes (involved in immune responses) originated from a single ancestral AIG1 domain-containing gene. Their diversification resulted from two successive gene duplication events that predated the divergence of green algae and land plants. The first duplication gave rise to the *IAN* lineage and the common ancestor of the *Toc34/Toc159* lineages, while the second duplication subsequently separated the *Toc34* and *Toc159* lineages.

However, the relationships among genes within each clade did not always reflect the established species phylogeny. For example, within the *IAN* lineage, genes from Poaceae formed a sister group to all other angiosperm, lycophyte, moss, and green algal genes, rather than grouping solely with other angiosperms. Similarly, in one clade of the *Toc159* lineage, three Poaceae genes (*Os05g05950*, *Os01g25450*, and *Os09g004020*) were placed at the base of the clade containing other Poaceae and eudicot genes. Such discrepancies can complicate the interpretation of evolutionary history. It has been reported that factors such as limited taxon sampling and the use of short sequence alignments in phylogenetic analyses can lead to artifacts like long-branch attraction, potentially skewing the results. Therefore, to reconstruct a more reliable evolutionary history for each lineage, we expanded our dataset by adding sequences from 12 additional species lacking whole-genome sequences (e.g., *Picea sitchensis*, *Elaeis guineensis*, and *Solanum tuberosum*; see Table S1 for details). We then constructed three separate alignments using full-length sequences to independently reconstruct phylogenetic trees for the *IAN*, *Toc34*, and *Toc159* genes (Figure S2A–C). This approach significantly improved the resolution and reliability of the trees, and the resulting gene relationships within each lineage became largely consistent with the known species phylogeny.

Based on these refined phylogenies, we found that the *IAN*, *Toc34*, and *Toc159* lineages have undergone markedly different evolutionary histories. Genes in the *IAN* lineage experienced numerous independent duplication events, occurring at the family, genus, and even species levels (Figure 2A and Figure S2A). For instance, the rice genes *Os02g35130* and *Os04g36030* and the sorghum genes *Sb04g022760* and *Sb06g017450* originated from a duplication event predating the divergence of Poaceae. The 14 IAN genes in *Arabidopsis thaliana* and the 12 in *A. lyrata* were generated through multiple recent within-genus and a few within-species duplications. Similar patterns of recent paralog expansion were observed in *Populus trichocarpa*, *Zea mays*, *Picea sitchensis*, and *Selaginella moellendorffii*.

The *Toc159* lineage underwent two large-scale gene duplication events prior to the divergence of angiosperms, giving rise to three major clades (Figure 2 andFigure S2B). Several more recent duplications subsequently occurred within each clade. In contrast, the *Toc34* lineage experienced only a few recent duplication events, identified in species such as *Populus trichocarpa*, *Solanum tuberosum*, *Vitis vinifera*, *Zea mays*, *Physcomitrella patens*, and possibly *Arabidopsis* (Figure 2 andFigure S2C). The frequent gene duplications at various evolutionary scales and the widespread distribution of recently duplicated genes across many plant species and lineages collectively explain the observed variation in copy number of AIG1 domain-containing genes in extant plants.

**Figure 3:**
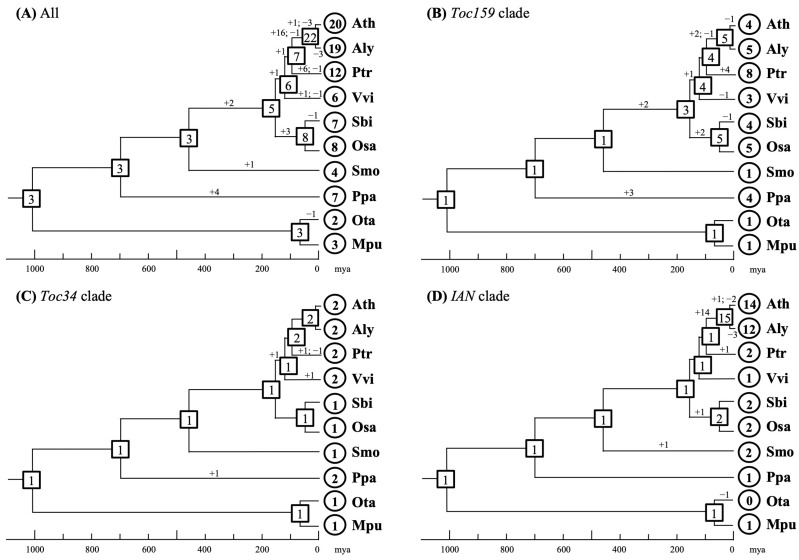
Evolutionary changes in *AIG1*-like gene copy number. Gene counts are shown for extant species (circles) and inferred ancestral nodes (rectangles). The plus (+) and minus (–) signs on branches indicate the number of genes gained or lost along each lineage since speciation.

To trace the dynamic changes in gene copy number throughout plant evolution, we estimated gene gains and losses by reconciling the gene trees with the species tree. Our analysis suggests that the most recent common ancestor (MRCA) of green plants possessed three AIG1 domain-containing genes, while the MRCA of angiosperms possessed five (Figure 3A). This indicates that the copy number variation among extant species is primarily the result of independent gene gains and losses. Furthermore, the extent of copy number variation differs considerably among the three lineages. The number of *Toc34* genes remained largely unchanged from green algae to angiosperms (Figure 3C), suggesting strong functional conservation in plastid protein import. In contrast, its partner, the *Toc159* lineage, experienced a dramatic increase in copy number in most species (Figure 3B). Notably, we infer that two additional *Toc159* genes were gained in the MRCA of angiosperms, alongside many independent gains and losses in other lineages. The presence of multiple *Toc159* copies in angiosperms could allow for more combinatorial interactions with Toc34 proteins, potentially increasing the specificity of preprotein recognition during plastid import. Unlike the *Toc34* and *Toc159* lineages, the *IAN* lineage maintained a low copy number (one or two genes) over a long evolutionary period, from the MRCA of green algae and land plants (~960 million years ago) to the MRCA of *Arabidopsis* and *Populus* (Figure 3D). However, a dramatic burst of gene duplication occurred specifically in the *Arabidopsis* lineage approximately 90 million years ago, expanding a single ancestral gene into 15 copies in the MRCA of the genus. Subsequent evolution in *A. thaliana* and *A. lyrata* involved additional gains and losses. This pattern of rapidly duplicating genes coexisting with evolutionarily conservative ones within the *IAN* lineage suggests highly unequal rates of gene duplication. In summary, although the overall birth rate for AIG1 domain-containing genes appears low, it varies significantly across different lineages and even among different plant groups within the same lineage.

### 2.3. Evolution of Genic Structure of Plant AIG1-Domain Containing Proteins

To gain further insight into the evolution of plant AIG1 domain-containing proteins, we mapped the exon–intron structure of each gene onto the corresponding phylogenetic tree (Figure 2B). Our results indicate that genes from different lineages exhibit distinct patterns of conservation and divergence in their structural organization. The *Toc34* lineage displays the most conserved gene structure. With the exception of the green algal genes *Ost31832* and *MicrCC31209*, all *Toc34* genes contain seven exons. Within this arrangement, exons 2 through 5 encode the AIG1 domain, while exon 6 encodes the C-terminal M domain (Figure 2B). The exon boundaries correspond to amino acid positions 33, 85, and 180 within the AIG1 domain alignment (Figure 1). Gene structures within the *Toc159* lineage are also relatively conserved, though more variable than in *Toc34*. Most *Toc159* genes possess one to three exons, except for those in *Physcomitrella patens*, which contain six (Figure 2B). Notably, the three major clades within this lineage exhibit distinct structural patterns. Genes in the clade containing *Toc132/Toc120* are intronless, the majority of genes in the *Toc90* clade contain a single intron, and genes in the *Toc159* clade have 0, 1, or 2 introns. Despite these variations, both the AIG1 and TM domains are consistently encoded by the longest exon in each gene.

In contrast, genes within the *IAN* clade possess complex and highly variable intron–exon structures, with exon numbers ranging from one to seven (Figure 2B). Unlike *Toc34* genes, the AIG1 domain in most *IAN* genes is encoded by exons 1, 2, and 3, with exon boundaries corresponding to amino acid positions 60 and 166 in the AIG1 domain alignment (Figure 1). Interestingly, we observed that recently duplicated gene pairs within the *IAN* lineage often diverge in their intron–exon structures. For example, the paralogs *AtIAN* (*At1g33870*) and *AtIAN2* (*At1g33880*) contain five and three exons, respectively. Similar structural divergence was found in other duplicated pairs, including *AtIAN11* (*At4g09930*) and *AtIAN12* (*At4g09940*), *Aly473550* and *Aly863596*, *Ptr249502* and *Ptr287977*, as well as *Smo24614* and *Smo446135*. Our analysis of intron–exon structures suggests that the gene architectures of the *IAN*, *Toc34*, and *Toc159* lineages have evolved independently following their divergence. Furthermore, the data imply that the AIG1 domain was likely encoded by a single exon in the ancestral plant *AIG1* gene. Subsequent lineage-specific intron insertions at different positions within this ancestral exon appear to have occurred after the separation of the *IAN*, *Toc34*, and *Toc159* lineages.

### 2.4. Gene Duplication Patterns in AIG1-like Genes

**Figure 4:**
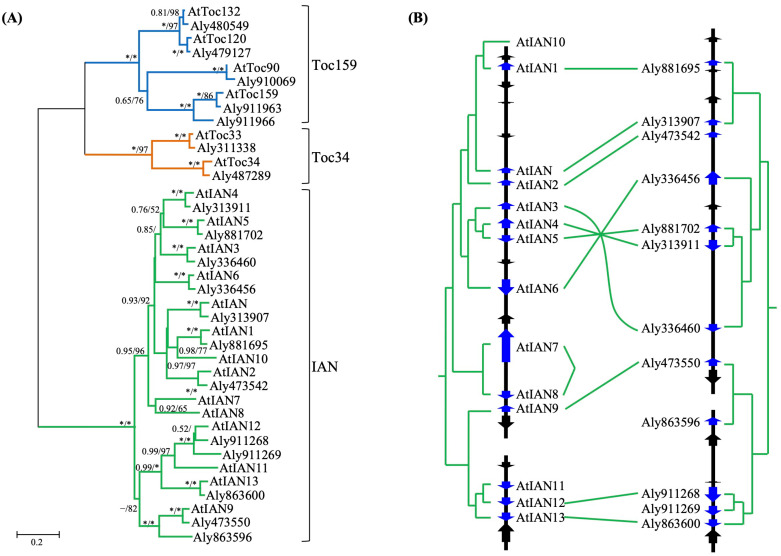
Evolution of *AIG1*-like genes in *Arabidopsis*. (**A**) Phylogenetic tree of *AIG1*-like genes in *A. thaliana* and *A. lyrata*, constructed using nucleotide sequences encoding the AIG1 domain. Tree reconstruction methods and branch support conventions follow Figure 1. (**B**) Genomic arrangement and transcriptional orientation of *IAN* clade genes. Genes are represented by arrows indicating direction of transcription. *AIG1*-like genes are colored blue, and other genes are shown in black. The diagram is drawn to scale. Paralogous genes in *A. thaliana* (**left**) and *A. lyrata* (**right**) are annotated according to the phylogenetic relationships shown in (**A**). Orthologous genes between the two species are connected by solid lines.

Previous studies have indicated that *IAN* genes in *A. thaliana* are organized into tandem arrays within certain chromosomal regions, suggesting that tandem duplication plays a significant role in their expansion. However, the mechanisms generating duplicate genes in other parts of the *IAN* lineage, as well as in the *Toc34* and *Toc159* lineages, remain unclear. By comparing the genomic locations of orthologous gene pairs, we detected clear microsynteny between the analyzed genomes, including instances of segmental inversion (Figure 4 and Figure S3).

Based on a well-resolved phylogenetic relationship (Figure 4A and Figure S2A), we inferred that among the 26 *IAN* clade genes identified in *A. thaliana* and *A. lyrata*, 24 (92.3%) were generated by tandem duplication, while the remaining 2 (7.7%) arose via dispersed duplication. This result indicates that tandem duplication is the predominant mechanism for the rapid expansion of *IAN* clade genes over short evolutionary timescales. Furthermore, we observed that newly formed tandem duplicated gene pairs often exhibit an altered orientation relative to the ancestral copy, resulting in “head-to-head” gene arrangements on the chromosome (e.g., the gene pair *AtIAN4* and *AtIAN5* in *A. thaliana*; Figure 4B). Combining phylogenetic and microsynteny evidence, we concluded that such “head-to-head” gene pairs originated at least three times in the MRCA of the two *Arabidopsis* species, and once independently in *A. thaliana* after their divergence (Figure 4B). Analysis of the intergenic regions between these “head-to-head” genes identified reverse complementary repeats, including palindromic sequences. In contrast to the typical pattern observed in many gene families (e.g., F-box, *SKP1*-like, and clade I MADS-box genes), where tandem duplicates usually share the same transcriptional orientation, our findings suggest that *IAN* clade genes in *Arabidopsis* may have expanded through a distinct tandem duplication mechanism. In addition to tandem duplication, we also identified one pair of *IAN* clade paralogous genes in *P. trichocarpa* that likely originated from a segmental duplication event, as they reside within clearly syntenic chromosomal blocks (Figure S3).

For *Toc159* clade, the general lack of introns in most members (except for those in moss) suggests that the ancestral gene of this clade may have arisen through an ancient retrotransposition event. However, typical hallmarks of retrogenes, such as a 3′ poly(A) tract and flanking short direct repeats, were not detected. This is likely because the putative retrotransposition event occurred in the distant past, and these sequence features have been obscured by subsequent mutations. Regarding gene expansion within this clade, segmental duplication also contributed to the increase in copy number of *Toc159* clade genes (Figure S3). For *Toc34* clade, however, the low number of copies that have persisted in most genomes left few traces of recent duplication events, with only one case of segmental duplication detected in *P. trichocarpa*.

## 3. Discussion

### 3.1. Evolutionary History of AIG1 Gene Family

Previous studies, noting the presence of *AIG1*-like genes in higher plants and vertebrates but not in prokaryotes [32,35,36,37], suggested that this gene family might have originated prior to the divergence of animals and plants. However, conclusive evidence supporting this hypothesis has been lacking, leaving the origin and evolution of *AIG1*-like genes an open question. Our comprehensive BLAST v2.16.0 searches identified numerous *AIG1*-like genes across a broad range of sequenced species, including protists (e.g., *Dictyostelium discoideum*, *Tetrahymena thermophila*), mollusks (e.g., *Lottia gigantea*), and cephalochordates (e.g., *Branchiostoma floridae*). Beyond angiosperms, we also identified these genes in green algae, mosses, ferns, and gymnosperms. These findings refute the earlier notion that AIG1-like proteins are exclusive to vertebrates and higher plants, and instead indicate their likely presence in early eukaryotes. Furthermore, the phylogenetic distribution suggests that the *AIG1*-like gene has been independently lost multiple times during evolution, explaining its absence in certain model organisms such as nematode (*Caenorhabditis elegans*), fruitfly (*Drosophila melanogaster*) and budding yeast (*Saccharomyces cerevisiae*) [37].

Based on our results, we propose a plausible scenario for the evolution of the *AIG1*-like gene family. The family likely originated very early in eukaryotic evolution, potentially associated with the emergence of eukaryotes themselves [38]. All plant *AIG1*-like genes share a common ancestor, whose protein product presumably contained only the core AIG1 domain. In the green plant lineage (after its divergence from red algae), this ancestral gene underwent two key duplication events, giving rise to the three major lineages (*Toc159 clade*, *Toc34* clade and *IAN* clade) identified in Figure 2A. Among these, the gene corresponding to *Toc159* clade may have arisen via retrotransposition. During subsequent evolution, proteins in *Toc34* clade and *Toc159* clade independently acquired distinct C-terminal domains—the M domain and the TM domain, respectively. The acquisition of these domains enabled their specific targeting to chloroplasts and their integration into the chloroplast protein import machinery, a key innovation in plastid biogenesis [39,40]. In contrast, proteins of *IAN* clade retained the ancestral AIG1 domain structure and were subsequently co-opted for functions in plant immune responses [33,35].

### 3.2. Phylogenetic Relationships and Evolutionary Dynamics of Plant Toc34, Toc159, and IAN Genes

In summary, different lineages of AIG1-like proteins exhibit distinct evolutionary patterns that correlate with their functional diversification. A key question arises: why do genes belonging to the same family diverge so markedly during evolution? A primary explanation lies in the presence, absence, and type of auxiliary domains associated with the core AIG1 domain. As established, all AIG1-like proteins share the conserved AIG1 domain, which serves as their defining sequence feature and enables a common biochemical role as GTP-binding molecular switches. This domain is essential for GTP binding and hydrolysis, forming the functional core of these proteins [41]. However, their participation in disparate biological processes, such as chloroplast protein import versus immune signaling, can be attributed to the acquisition of distinct functional modules beyond the AIG1 domain [42]. For instance, during evolution, the ancestor of the *Toc34* lineage acquired a C-terminal M domain, which is indispensable for membrane anchoring. Conversely, the ancestor of the *Toc159* lineage acquired a transmembrane (TM) domain, committing it to function as an integral membrane protein [17,43]. Thus, the gain of different C-terminal domains was a critical driver of functional divergence following gene duplication.

This study has established the evolutionary framework for the *AIG1* gene family in plants and elucidated patterns of duplication and diversification within each lineage. We demonstrate that the *Toc34* lineage has maintained relatively stable copy numbers, suggesting functional constraint. In contrast, the *Toc159* lineage has undergone more dynamic “birth-and-death” evolution [44], with significant fluctuations in gene number. The *IAN* lineage presents a mosaic pattern, where rapidly duplicating genes coexist with evolutionarily conservative ones [45]. A notable case of rapid proliferation is observed in the eudicot lineage, where *IAN* gene copy number increased from a single copy in the MRCA of Brassicaceae and Salicaceae to at least 15 copies in the MRCA of the genus *Arabidopsis* within a relatively short evolutionary span of ~90 million years. Moreover, even during the brief divergence period of 5–6 million years between *A. thaliana* and *A. lyrata*, their *IAN* gene repertoires continued to undergo dynamic turnover. *A. thaliana* experienced one gain and two losses, while *A. lyrata* lost three copies. Such a pronounced increase in gene birth rate raises the question of what functional or adaptive pressures drove the recruitment and retention of so many paralogs in this specific lineage. One plausible hypothesis is that members of rapidly duplicating clades have been selected to enhance the plant’s capacity to respond to diverse environmental or pathogenic stimuli [46,47]. Our findings reveal a strong correlation between evolutionary pattern and gene function, providing a testable framework for future research. The observed changes in gene copy number, evolutionary rate, and expression appear non-random and are hypothesized to be constrained by functional requirements, although this awaits direct experimental validation.

Furthermore, we found that rapidly duplicating genes, particularly within the *IAN* lineage, are predominantly generated by tandem duplication. This pattern aligns with observations in other large gene families, such as the F-box superfamily, MADS-box genes, and *SKP1*-like genes [48,49,50]. This prevalence can be explained by the mechanistic propensity for tandem duplication to occur frequently during plant evolution [51]. Compared to segmental duplication or retrotransposition, tandem duplication offers a more rapid pathway to generate multiple gene copies within a short evolutionary timeframe, thereby enabling plants to meet immediate and specific functional demands [52].

### 3.3. Mechanism Underlying the Generation of Head-to-Head Tandem Duplicated Genes

Previous studies have established that tandemly duplicated genes typically share the same transcriptional orientation along a chromosome [52,53]. In this study, however, we observed that many newly formed tandem duplicates of *IAN* clade *AIG1*-like genes in *A. thaliana* and *A. lyrata* are arranged in a head-to-head orientation relative to the ancestral copy. This suggests a distinct mechanistic basis for their generation.

**Figure 5:**
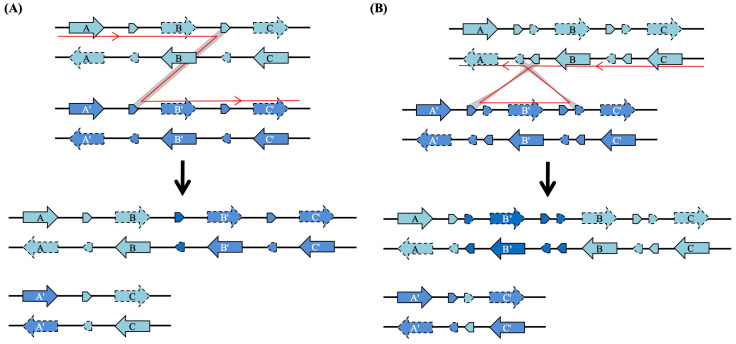
Model for the generation of tandemly duplicated genes. (**A**) The conventional model: Tandem duplication typically occurs via unequal crossing-over between direct repeats flanking a gene on homologous chromosomes. Recombination between identical DNA strands produces duplicated genes with the same transcriptional orientation. (**B**) Proposed model for head-to-head duplication: The presence of inverted repeat sequences (palindromes) upstream of the progenitor gene allows unequal crossing-over to occur between complementary DNA strands. This process generates duplicated genes in a head-to-head orientation. The expansion of head-to-head *AIG1*-like genes may have occurred via this mechanism. The red line indicates the direction of crossing-over.

The classical model of tandem duplication involves unequal crossing over during homologous recombination, typically mediated by flanking direct repeat sequences [54] (Figure 5A). In this scenario, recombination occurs between the same DNA strands (e.g., both forward strands) of homologous chromosomes, invariably preserving the original orientation of the duplicated segment [55]. In contrast, the head-to-head arrangements we identified are associated with the presence of reverse complementary repeats, including palindromic sequences, within the intergenic regions where duplication occurred [56,57] (Figure 5B). These structural features could facilitate unequal crossing over between opposite DNA strands (e.g., forward and reverse strands) of homologous chromosomes [58]. Recombination at such sites would invert the orientation of the newly duplicated copy relative to its progenitor, thereby producing the observed head-to-head gene pairs [57,59,60]. The consistent presence of these reverse complementary or palindromic sequences in all relevant intergenic regions supports a high probability for this inversion-mediated duplication mechanism. Furthermore, head-to-head gene pairs often share a single intergenic promoter region, which may allow for their coordinated co-expression, a functional implication that warrants experimental validation [59,60,61]. While this mechanism appears prominent in the expansion of *AIG1*-like genes, its prevalence in other gene families remains to be determined through broader genomic analyses.

### 3.4. The Importance of Copy Number Variation and Sequence Changes in Plant Evolution

In research over recent decades, evolutionary studies have predominantly focused on the contribution of nucleotide and amino acid sequence changes [62,63]. However, sequence divergence alone cannot account for all evolutionary phenomena. Variation in the copy number of gene families represents another fundamental evolutionary mechanism [64,65]. For example, for the *AIG1*-like gene family studied here, copy numbers range from 1 to 20 across the 11 investigated species. A notable dynamic is observed even between the closely related species *A. thaliana* and *A. lyrata*, where only 72.7% (16 of 22) of *AIG1*-like genes maintain a strict one-to-one orthologous relationship. This indicates that over a quarter (6 of 22) of these genes were gained or lost within the last 5–6 million years, a pattern consistent with frequent copy number changes reported in other families, such as MADS-box genes in *Arabidopsis* and various sensory receptor genes [66,67]. These findings underscore those changes in gene copy number is a major mechanism for reshaping genomic content [64,65]. Consequently, estimating species divergence based solely on the sequence similarity of orthologs is neither strictly accurate nor sufficient. Orthologous sequence divergence captures only one dimension of genomic change, while gain, loss, and copy number variation of genes represent a critical and complementary layer of evolutionary innovation. A comprehensive understanding of plant evolution therefore requires the integrated analysis of both sequence-level changes and structural genomic variation. However, it is important to acknowledge a limitation inherent in our sampling strategy. While our study encompasses major plant lineages, the evolutionary inferences are drawn from a set of 11 representative species. The limited sampling among early-diverging lineages (e.g., green algae and bryophytes) may constrain the resolution of evolutionary dynamics during the early stage of diversification. Therefore, the ancestral gene numbers estimated here should be interpreted with caution. Future studies incorporating a broader taxon sampling will be essential to verify and refine the patterns observed in further studies.

## 4. Materials and Methods

### 4.1. Sequence Retrieval and Domain Analysis

To identify homologs of Toc34, Toc159, and IAN proteins, we performed Hidden Markov Model (HMM) searches against the predicted proteomes of 13 organisms with fully sequenced genomes [68], including ten plant species and three non-plant species used as outgroups (see Table S1). The proteomes and corresponding genome data were obtained from publicly available databases, including Phytozome (https://phytozome-next.jgi.doe.gov, accessed on 12 January 2025), the National Center for Biotechnology Information (NCBI, http://www.ncbi.nlm.nih.gov, accessed on 15 January 2025), The Arabidopsis Information Resource (TAIR, http://www.arabidopsis.org, accessed on 15 January 2025), and EnsemblProtists (http://protists.ensembl.org, accessed on 18 January 2025). The HMM profile for the AIG1 domain (PF04548, build 2.3.2) was downloaded from the Pfam database (http://pfam.xfam.org, accessed on 22 January 2025). The initial dataset was filtered to remove sequences lacking the AIG1 domain using the online tools available on the SMART and Pfam websites. Additionally, AIG1-like protein sequences were retrieved from 12 additional plant species of phylogenetic importance by performing BLASTP v2.16.0 searches against the NCBI non-redundant protein database (nr) (Table S1). Multiple known AIG1-like proteins were used as queries to ensure comprehensive retrieval. An E-value cutoff of 10^−5^ was applied in both the HMM and BLASTP searches.

For clarity in presentation, genes from *Arabidopsis thaliana*, *Oryza sativa* ssp. *japonica*, and *Sorghum bicolor* are referred to by their standard annotated locus identifiers (e.g., *Os01g25450* for rice). For genes from other species, we generated names by combining the first letter of the genus name and the first two letters of the specific epithet, followed by the sequence identifier (e.g., the *Arabidopsis lyrata* sequence with accession number 311,338 was named *Aly311338*). Functionally characterized genes in *A. thaliana* are referred to by their established names (e.g., *Toc33*, *Toc34*, *Toc159*, and *IAN*).

Beyond the core AIG1 domain, Toc34 proteins possess a conserved C-terminal M domain, and Toc159 proteins contain a characteristic transmembrane (TM) domain. The M domain in Toc34-like proteins was identified using the domain-search tools on the SMART and Pfam websites, where it is designated as DUF3406 (PF11186). The TM domain of Toc159 is not represented by a specific model in these databases. Therefore, we employed the MEME (Multiple Expectation Maximization for Motif Elicitation) suite (http://meme-suite.org, accessed on 25 February 2025) to identify conserved motifs within the C-terminal region of Toc159 and related proteins, corresponding to the TM domain.

### 4.2. Sequence Alignment and Phylogenetic Analysis

Protein sequences were initially aligned using the hmmalign program from the HMMER v3.4 to generate a preliminary matrix [69]. To ensure a robust alignment, particularly for the conserved AIG1 domain region, this preliminary matrix was realigned using MAFFT v7.505 with default parameters and subsequently refined manually in GeneDoc v2.6.0.2 [70,71]. As noted in the Introduction, Toc34, Toc159, and IAN homologs share the AIG1 domain but possess highly divergent C-terminal regions. Therefore, phylogenetic analysis involving all clades was conducted using an alignment restricted to the AIG1 domain region. For individual phylogenetic analyses of the *Toc34*, *Toc159*, and *IAN* clades, full-length sequence matrices were employed. Due to the presence of highly variable regions within both the AIG1 domain and the C-terminal segments, not all amino acid sites were included in the final matrices. To objectively assess alignment quality and retain only reliably aligned positions, the column score for each site was calculated in Clustal W2 [72]. Based on empirical practice in phylogenetic analyses and preliminary filtering tests, sites with a column score greater than 12 were retained for subsequent tree reconstruction, thereby improving the robustness of the phylogenetic inference.

In addition to protein-based matrices, a corresponding DNA alignment of all *AIG1*-like sequences from *A. thaliana* and *A. lyrata* was generated for phylogenetic analysis using the aa2dna script. To obtain well-supported phylogenetic hypotheses, trees were reconstructed using three independent methods: Neighbor-Joining (NJ), Maximum Likelihood (ML), and Bayesian Inference (BI). Neighbor-Joining (NJ) analyses were performed in MEGA 12 using the p-distance model [73], with pairwise deletion of gaps and default assumptions of homogeneous substitution patterns and rates among lineages and sites. Branch support was assessed with 1000 bootstrap replicates. Maximum Likelihood (ML) analyses were conducted with IQ-TREE [74]. For protein matrices, the WAG substitution model was used, with the proportion of invariable sites and the gamma distribution shape parameter optimized automatically. A BIONJ tree served as the starting topology. For DNA matrices, the HKY model, determined as optimal by ModelFinder [75], was applied. Other parameters matched those used for protein ML analyses. Nodal support for all ML trees was evaluated using 100 bootstrap replicates. Bayesian Inference (BI) analyses were performed using MrBayes 3.2.7a [76]. Four independent Markov chain Monte Carlo (MCMC) chains were run for 1,000,000 generations, sampling one tree every 1000 generations from a random starting tree. The first 50,000 generations were discarded as burn-in to ensure stationarity. Posterior probabilities were used to estimate branch support. The WAG and HKY substitution models were applied to protein and DNA matrices, respectively, consistent with the ML analyses.

### 4.3. Determination of the Duplication Types

New genes arise primarily through three mechanisms: tandem duplication, segmental (or whole-genome) duplication, and retroposition. To elucidate which of these mechanisms contributed to the expansion of the *AIG1* gene family, we assessed their relative contributions across all sampled plant genomes. Tandem duplicates were identified based on two criteria: (1) genes formed a closely related clade in the phylogenetic tree, and (2) genes were located on the same chromosome with no more than 20 intervening genes between them. Segmental duplicates were identified by searching for systemic blocks using the Plant Genome Duplication Database (PGDD) [77]. Genes located within systemic blocks were considered products of segmental duplication. For genes not accounted for by the above mechanisms, we evaluated whether they originated via retroposition. A gene was considered a potential retrogene if it met the following criteria: (1) lacked introns, (2) contained a poly(A) tract at its 3′ end, (3) was flanked by short direct repeats, and (4) was located on a chromosome different from its putative donor gene locus.

## Data Availability

The original contributions presented in this study are included in the article/Supplementary Materials. Further inquiries can be directed to the corresponding author.

## Supplementary Materials

The following supporting information can be downloaded at: https://www.mdpi.com/article/10.3390/plants15020301/s1, Table S1: List of *AIG1*-like genes analyzed in this study. Figure S1: Multiple-sequence alignment of the (A) TM domain and (B) M domain. Representative sequences containing these domains are shown. Conserved residues are highlighted. Figure S2: Phylogenetic trees of the (A) *Toc159*, (B) *Toc34*, and (C) *IAN* clades. To mitigate potential long-branch attraction, sequences from 12 additional species were included alongside the core set of 90 AIG1 proteins from 11 representative species. Trees were constructed using Neighbor-Joining (NJ), Maximum Likelihood (ML), and Bayesian Inference (BI) methods. The topology shown is from the BI analysis. Branch support is indicated as follows: asterisks (*) denote nodes with a posterior probability (PP) of 1.00 in BI or 100% bootstrap support (BS) in ML/NJ analyses; dashed lines (–) indicate nodes with PP < 0.50, BS < 50%, or topological conflict between methods. Scale bars represent the number of substitutions per site. Figure S3: Microsynteny analysis of *AIG1*-like genes in *Populus trichocarpa*.
