# Supplementary material for: Evolution of Plant AIG1-like Proteins: Different Modes of Sequence Divergence and Their Contributions to Functional Diversification
Source: Plants (Basel). 2026 Jan 19;15(2):301. doi: 10.3390/plants15020301 (PMC12845241; doi:10.3390/plants15020301)
Supplement: Supplementary file 1 [file plants-15-00301-s001.zip › plants-4096190-supplementary/Supplementary Figure S1-3.pdf]

(A) TM Domain

Toc159

```
                        *        20         *        40         *        60         *        80         *       100         *       120
AtToc132   : FDSDNPTHRYRYLDSSNQWLVRPVLETHGWDHDIGYEGVNAERLFVVKEKIPISVSGQVTKDKKDANVQLEMASSVKHGEGK-STSLGFDMQ-TVGKELAYTLRSETRFNNFRRNKAAAGLSV : 121
Vvi36085   : FDSDNPTHRYRYLDSSNQWLVRPVLETHGWDHDVGYEGINVERVFAIKDKIPVSFSGQVTKDKKDANLQMEIASSVKHGEGK-ATSVGFDMQ-TVGKDMAYTLRSETRFCNFRKNKATAGLSI : 121
Ptr713242  : FDSDNPTHRYRYLDTSNQWLVRPVLETHGWDHDVGYEGINVERLFVVKDKIPISFSGQVTKDKKDANVQMELASSLKYGEGK-ATSLGFDMQ-TVGKDLAYTLRSETRFSNFRKNKATAGLSV : 121
Ptr200841  : FDSDNPSHRYRYLDNSNQWLVRPVLDTHGWDHDVGYEGINVERLFVVKDKIPVSFSGQVTKDKKDANVQMELASSIKHGEGK-STSLGFDMQ-TLGKDLGYTLHSETRFINFRKNKATAGLSV : 121
Os10g40110 : FDSDHPSHRYRFLDTPSEWLVRPVLETQGWDHDVGYEGLNVERLFAVKGKVPLSVSGQLSKDKKDCSLQMEVASSLKHGEGK-TTSLGLDLQ-SVGKDMAYTLRGESRFKNFRRNNTAAGISA : 121
AtToc19    : FDSDFPAHRYRCVSAGDQWLVRPVYDPQGWDRDVGFDGINIETAAKINRNLFASATGQVSRDKQRFTIQSETNAAYTRNFREQTFSVAVDLQ-SSGEDLVYSFQGGTKLQTFKHNTTDVGVGL : 122
Ptr578875  : FDSDCTIHKYRCLVTSDQWLVRPVLDPHGWDHDVGFDGVNLETAIEIRRNVYASITGQMSKDKQDFSIHSECAAAYA-DPRGQTYSAALDVQTSSGKGMIYTVHSNTKLRNLKQNVIECGVSL : 122
Vvi06412   : FDSDCPAHRYRCLVMSDQWLVRPVLDPHGWDHDVGFDGINLETTMDLKGNLIASVTGQMSKDKQDFSIQSECAAVYT-DPRGPNYFVGLDVQ-SAGKDLIYTVHSNTKMRNLKHNLTECGFSM : 121
Os12g09570 : FDSDYPVHRYRYLIT-DDMVFRPVLDPQGWDHDIGFDGINFEVSQDLEKNTTASIAGQMRKDKEDLYIQSECSVSYS-GKNGRSLIGSMNMQ-TANKDLVCTVNGDARFHNLPWNTTGGGISI : 120
AtToc159   : FDSDNSAYRYRYLEPTSQLLTRPVLDTHGWDHDCGYDGVNAEHSLALASRFPATATVQVTKDKKEFNIHLDSSVSAKHGENG-STMAGFDIQ-NVGKQLAYVVRGETKFKNLRKNKTTVGGSV : 121
Ptr278713  : FDSDNPSYRYRALEPTSQFLMRPVLDSHGWDHDCGYDGVSLERNLAVAGQFPGAFAVQITKDKKDFNIHLDSSVCAKHGENG-STMAGFDIQ-NVGRQLAYILRSETKFKNFKMNKTSAGISF : 121
Vvi27973   : FDCDNPAYRYRFLEPTSQFLARPVLDTHGWDHDCGYDGVNLEQSLAILGQFPAAVSVQVTKDKKEFNIHLDSSAAAKHGENG-SSMAGFDIQ-NIGKQLAYILRGETKFKILKKNKTAAGFSV : 121
Os05g05950 : FDCDNPTYRYRFLEPTSTVLARPVLDAHGWDHDCGYDGVSVEETLALLNKFPANMAVQVTKDKKEFSIHLDSSISAKLGEDA-SSLAGFDIQ-TVGRQLAYILRGETKFKNIKKNKTTGGFSV : 121
Os03g61890 : FDSDDPAYRYRCLDPKPNFLVRAITNPDGWDHDCGFDGVSLQYSLDAANAFPASLWVQVNKDKRESTIHLVSSISAKHRENV-SSLAGFDIQ-TIMDQLAYTLRGESKFKNSKKNTTTGGLSM : 121
Smo170624  : FDSDNPVHRYRYLETASQWLVRPVMDLHGWDRDSGYDGFNMERMFTYG-KIPASLSGQVTKDKKEANVTLECAASMKHGEGK-VSQAGFDVQ-TVGKDFSYTLRSETRFSNFKKNKTIAGLAM : 120
Ppa216964  : FDSDNPTHRYRYLETANQWLVRPVLETHGWDHDAGYDGFNVEKMFVVKEKIPASVSGQVTKDKKEAQVNFEAAASLRHGEGK-VTLTGFDVQ-TIGKDLAYTVRAETRFNNFKRNKTTAGVTA : 121
Ppa216050  : FDSDNPTHRYRYLETANQWLVRPVLETHGWDHDAGYDGFNVEKMFVVKNKIPASISGQVTKDKKESQVNFEAAASLKHGEGK-VTLTGFDVQ-TIGKDLAYTLRAETRFNNFKRNKTTAGVTA : 121
```

Toc159

```
                        *       140         *       160         *       180         *       200         *       220         *       240
AtToc132   : THLGDSVSAGLKVEDKFIASKWFRIVMSGGAMTSRGDFAYGGTLEAQLRDKDYPLGRFLTTLGLSVMDWHGDLAIGGNIQSQVPIGRSSNLIARANLNNRGAGQVSVRVNSSEQLQLAMVAI : 243
Vvi36085   : TALGDAITAGLKLEDKLIVNKRIRLVMTGGAMTGRGDVAYGGSLEATLRDKDHPLGRSLSTLGLSIMDWHGDLAIGCNIQSQIPIGRFTNMIGRVNLNNRGAGQVSIRLNSSEQLQIALIGL : 243
Ptr713242  : TLLGDVLSAGVKVEDKLIAGKRLQMVMSGGAMAGRGDVAYGGSLEVQLRDKDYPLGRSLSTLGLSVMDWHGDLAIGCNVQSQIPIGRSTNLIGRANLNNRGAGQISIRVNSSEQLQLALISL : 243
Ptr200841  : TLLGDALSAGVKVEDKLIANKRFQMVMSGGAMTGRGDVAYGGSLEAQLRDEDYPLGRSLSTLGLSVVDWHGVLAIGCNIQSQVPIGRSTNLIARANLNNKGAGQLSIRINSSEHLQIALAGL : 243
Os10g40110 : TLLGDSVSAGVKVEDKLVVNKQLRVLVSGGAMSGRGDVAYGGRLEATLKDKDYPIGRMLSTIALSVVDWHGDLAVGCNIQSQIPAGRASNLIGHANLSNKGTGQVGIRLNSSEHLEIALIAL : 243
AtToc19    : TSFGGKYYVGGKLEDTLLVGKRVKLTANAGQMRGSGQTANGGSFEACIRGRDYPVRNEQIGLTMTALSFKRELVLNYGLQTQFRPARGTNIDVNINMNNRKMGKINVKLNSSEHWEIALISA : 244
Ptr578875  : TSYDNKYYVGAKLEDTILVGKRLKVVVNAGQMRGPEQVAYGGTLEATLKGGDYPVRDDRISLSMSALSFKNEMVLGGGFQSEFRPIRGMRMAVNANLNSQNMGQVNIKISSSVHIEIALVAV : 244
Vvi06412   : TSFRNKYCVGAKLEDTISIGKRLKFVMNVGQMGGLEQVAYGGSFGATLRGRDYPARKDSASLNMALLSLNKEMVMSGSIQSDFRSSRGTRMSINANLNSRKMGQICIKTSSSEHMEIALVAF : 243
Os12g09570 : TKFGSKYFTGAKLEDSITVGRRVQLVGNAGRMVGCGQVAHGGGLEMTFRGKDYPVREESITVAATALSFEKETVISTNLQSDFRMGRGSKVSVSANLNSRNLGRFCVKTSTSDHSEIALVAA : 242
AtToc159   : TFLGENIATGVKLEDQIALGKRLVLVGSTGTMRSQGDSAYGANLEVRLREADFPIGQDQSSFGLSLVKWRGDLALGANLQSQVSVGRNSKIALRAGLNNKMSGQITVRTSSSDQLQIALTAI : 243
Ptr278713  : TVLGENVATGLKIEDQIAVAKRLALVGAAGAVRSGGDTAYGANFEVCLKSKDFPIEKDQSTLGLSLMKWRGDLGLMANLQSQFSIGRNSKMAVRVGMNNKRSGQVTIKTSSSE-MQVALIAI : 242
Vvi27973   : TFLGENVATGFKVEDQFTLGKRLVLAGSTGTVRCQGDAAYGANLEVRLREADFPIGQDQSTLGLSLVKWRGDLALGANLQSQFSIGRSSKMAVRVGLNNKLSGQITVKTSSSEQLQIALVGI : 243
Os05g05950 : TFLGDIVATGLKVEDQLSLGKRLALVASTGAMRAQGDTAYGANLEARLKDKDYPIGQSLSTLGLSLMKWRRDLALGANLQSQFSIGRGSKMVVRLGLNNKLSGQITVRTSTSEQVQIALLGL : 243
Os03g61890 : TFLGDTMVTGAKFEDKLSVGDRLTLLANTGAVSIRGDTAYGVNMEATLREKDYLMGQDLAILGASLVRWHKEWSMAAKLDSQFSMGRASNVAVHVDLTNKLTGRVSIKANTSEQLKIALLGV : 243
Smo170624  : TMIGDTLAYGMKIEDRLMIGRRVKVVMNGGALTGKGDTAYGGTVEISLRDKDFPLGRSLSTCGLSVMNWHGDMALGGNLQSQFMVG-KTMVVARGNLNNRGAGQITIRLSSSEQLQIALIGV : 241
Ppa216964  : TYLNDTIAAGVKLEDRVLIGKRVKLVVNGGVLTGKGDKAYGGSLEATLRGKEYPLSRTLSTLGLSVMDWHGDLAIGGNLQSQFMVG-KTMMVGRANLNNRGSGQVSIRASSSEQLQMVLIGI : 242
Ppa216050  : TYLNDTIAAGVKLEDRILIGKRVKMVVNGGVLTGKGDKAFGGSLEATLRGKEYPLSRTLSTLGLSVMDWHGDLAIGGNLQSQFMVG-KTMMVGRANLNNRGSGQVSIRASSSEQLQMVLIGI : 242
```

(B) M Domain

Toc34

```
                        *        20         *        40         *
AtToc33    : ITDVATNQRKAIHVDKKMVDGSYSDDKGKKLIPLIIGAQY-LIVKMIQGAIRNDI : 54
Ptr834816  : IVGVATNGKKSLLVDEKLVNGSESNDRGKIFIPLILGVQW-LLVKWIQREIKKDI : 54
AtToc34    : ITEISFNGNKAIHVDKKLVEGPNPNERGKKLIPLMFAFQYLLVMKPLVRAIKSDV : 55
Vvi14090   : IIDLVSNGSKAILVDKKLIEGPNPNKRGKFLIPLIVAFQY-LVVKQIQRTIKDDV : 54
Os03g13730 : ITTVVSNGSKSIHVDQKLIDGPNPNNRWKMFIPLILMVEYFLVVKGIRRAIHADI : 55
Smo160107  : LVEVVTNEHPSLLVDKKLIEGPNANNRGKLWIPLVLLAQYLLVVRPIRKAIENDI : 55
Ppa125298  : IAEVATGATPSIHVDQKLIDGPNANGKGKWWIPLIILAQYFLIVRPIRSAIEKDL : 55
```

Figure S1. Multiple sequence alignment of the (A) TM domain and (B) M domain. Representative sequences containing these domains are shown. Conserved residues are highlighted.

(A) *Toc159* clade

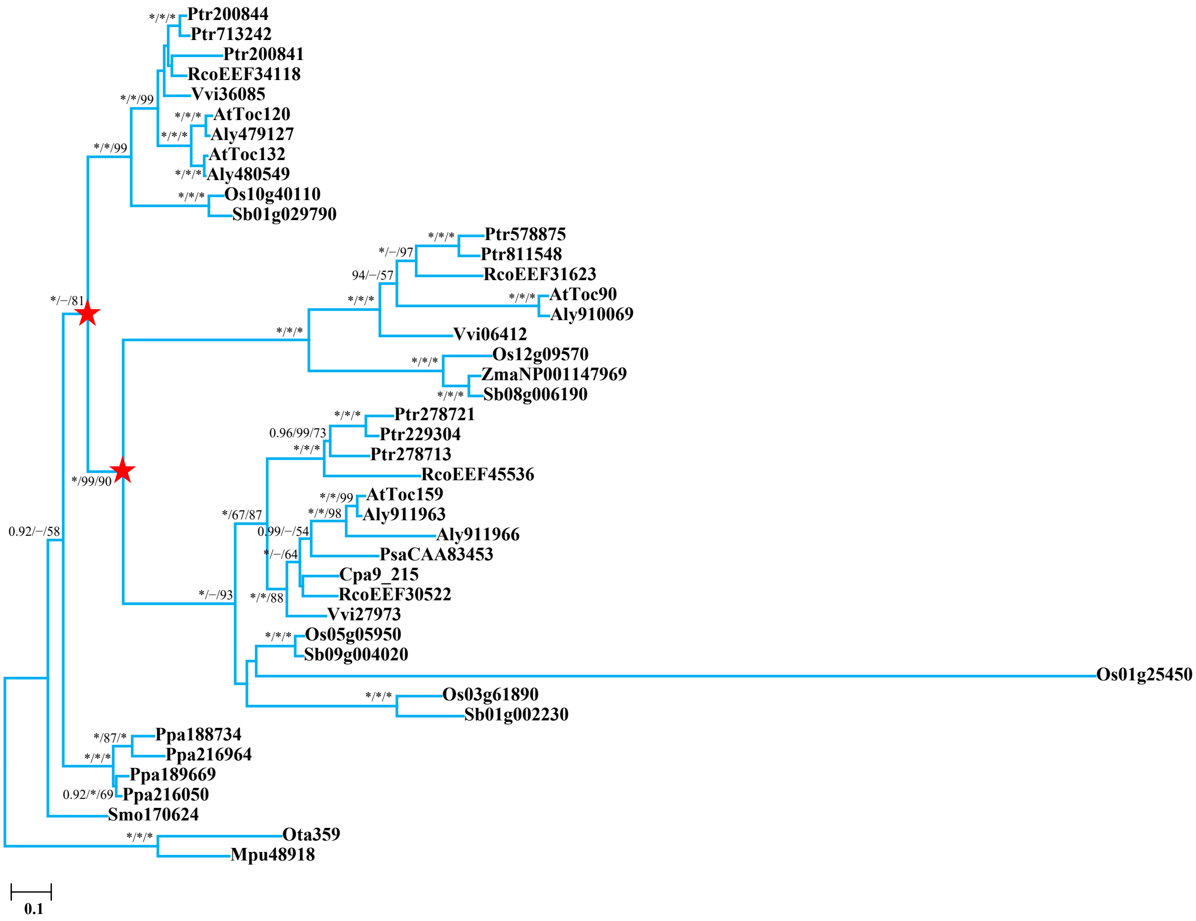

(B) *Toc34* clade

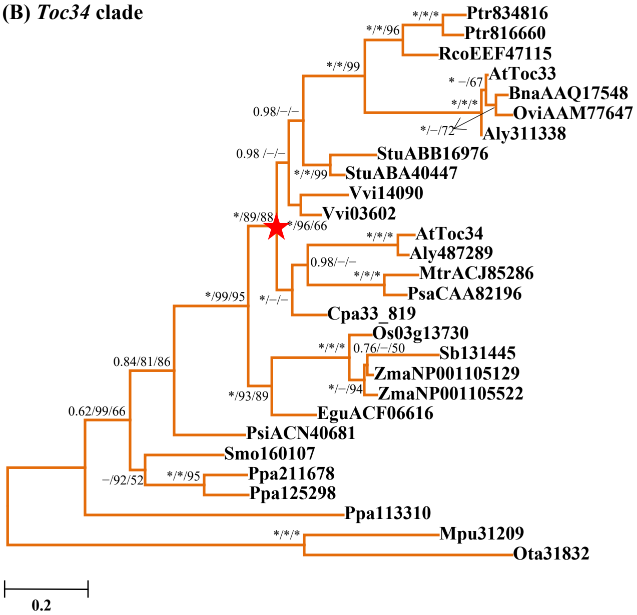

(C) ***IAN*** **clade**

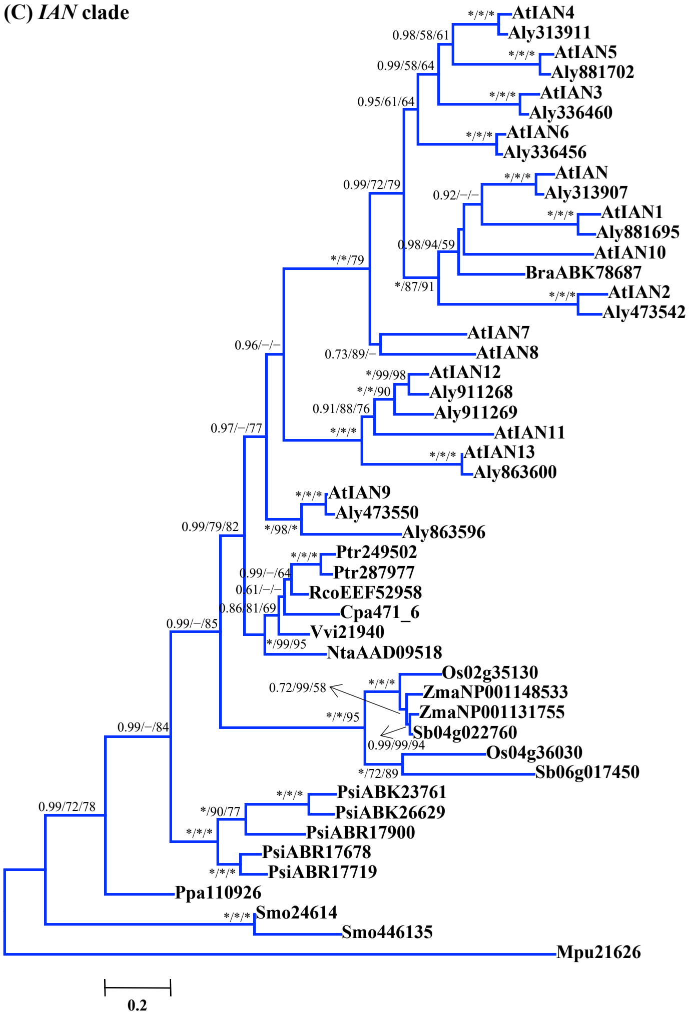

Figure S2. Phylogenetic trees of the (A) *Toc159*, (B) *Toc34*, and (C) *IAN clades*. To mitigate potential long-branch attraction, sequences from 12 additional species were included alongside the core set of 90 AIG1 proteins from 11 representative species. Trees were constructed using Neighbor-Joining (NJ), Maximum Likelihood (ML), and Bayesian Inference (BI) methods. The topology shown is from the BI analysis. Branch support is indicated as follows: asterisks (*) denote nodes with a posterior probability (PP) of 1.00 in BI or 100% bootstrap support (BS) in ML/NJ analyses; dashed lines (–) indicate nodes with PP < 0.50, BS < 50%, or topological conflict between methods. Scale bars represent the number of substitutions per site.

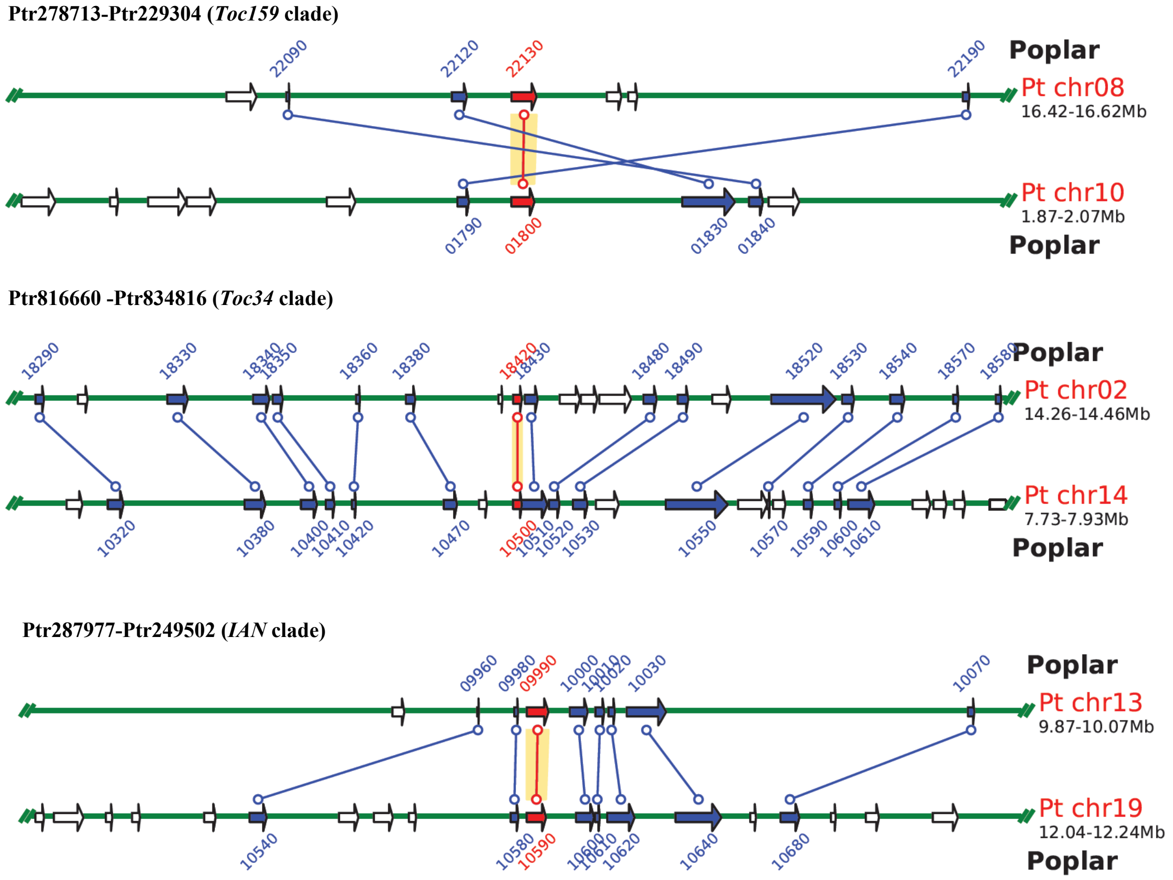

Figure S3. Microsynteny analysis of *AIG1*-like genes in *Populus trichocarpa*.
