# Supplementary material for: Evolution of Plant AIG1-like Proteins: Different Modes of Sequence Divergence and Their Contributions to Functional Diversification
Source: Plants (Basel). 2026 Jan 19;15(2):301. doi: 10.3390/plants15020301 (PMC12845241; doi:10.3390/plants15020301)
Supplement: Supplementary file 1 [file plants-15-00301-s001.zip › plants-4096190-supplementary/Supplementary Table S1.pdf]

Species	Database	Num	Gene name	Nomenclature	Accession number	Location
**Model species**						
Arabidopsis lyrata	Phytozome	19	Aly311338	fgenesh1_pm.C_scaffold_1000118	311338	scaffold_1:571279-572890
			Aly313907	fgenesh1_pm.C_scaffold_1002687	313907	scaffold_1:15799130-15800273
			Aly313911	fgenesh1_pm.C_scaffold_1002691	313911	scaffold_1:15821792-15823812
			Aly336456	fgenesh1_pg.C_scaffold_1003021	336456	scaffold_1:15808990-15811489
			Aly336460	fgenesh1_pg.C_scaffold_1003025	336460	scaffold_1:15837743-15839092
			Aly473542	fgenesh2_kg.1_3496_At1G33880.1	473542	scaffold_1:15801599-15802559
			Aly473550	fgenesh2_kg.1_3504_At1G33970.4	473550	scaffold_1:15844037-15845872
			Aly479127	fgenesh2_kg.3_1828_At3G16620.1	479127	scaffold_3:7047066-7050462
			Aly480549	fgenesh2_kg.3_3250_At2G16640.1	480549	scaffold_3:21344502-21348890
			Aly487289	fgenesh2_kg.6_423_At5G05000.1	487289	scaffold_6:1741856-1744168
			Aly863596	Al_scaffold_0006_3105	863596	scaffold_6:19837426-19838788
			Aly863600	Al_scaffold_0006_3109	863600	scaffold_6:19856258-19857631
			Aly881695	Al_scaffold_0001_3462	881695	scaffold_1:15788111-15789072
			Aly881702	Al_scaffold_0001_3469	881702	scaffold_1:15818953-15820308
			Aly910069	scaffold_602126.1	910069	scaffold_6:8588808-8591612
			Aly911268	scaffold_603325.1	911268	scaffold_6:19850013-19852882
			Aly911269	scaffold_603326.1	911269	scaffold_6:19853541-19855264
			Aly911963	scaffold_604020.1	911963	scaffold_6:23642916-23647671
			Aly911966	scaffold_604023.1	911966	scaffold_6:23673498-23676811
Arabidopsis thaliana	TAIR	20	Toc33	At1g02280	At1g02280	At1g02280
			AtIAN1	At1g33830	At1g33830	At1g33830
			AtIAN	At1g33870	At1g33870	At1g33870
			AtIAN2	At1g33880	At1g33880	At1g33880
			AtIAN3	At1g33890	At1g33890	At1g33890
			AtIAN4	At1g33900	At1g33900	At1g33900
			AtIAN5	At1g33910	At1g33910	At1g33910
			AtIAN6	At1g33930	At1g33930	At1g33930
			AtIAN7	At1g33950	At1g33950	At1g33950

			AIG1, AtIAN8	At1g33960	At1g33960	At1g33960
			AtIAN9	At1g33970	At1g33970	At1g33970
			Toc132	At2g16640	At2g16640	At2g16640
			AtIAN10	At2g26820	At2g26820	At2g26820
			Toc120	At3g16620	At3g16620	At3g16620
			Toc159	At4g02510	At4g02510	At4g02510
			AtIAN11	At4g09930	At4g09930	At4g09930
			AtIAN12	At4g09940	At4g09940	At4g09940
			AtIAN13	At4g09950	At4g09950	At4g09950
			Toc34	At5g05000	At5g05000	At5g05000
			Toc90	At5g20300	At5g20300	At5g20300
Homo sapiens	NCBI	7	HsaGIMAP7		NP694968	
			HsaGIMAP4		NP060796	
			HsaGIMAP5		NP060854	
			HsaGIMAP2		NP056475	
			HsaGIMAP1		NP570115	
			HsaGIMAP6		NP078987	
			HsaGIMAP8		NP783161	
Micromonas pusilla CCMP1545	Phytozome	3	Mpu21626	e_gw1.12.339.1	21626	scaffold_12:342535-343050
			Mpu31209	estExt_Genewise1Plus.C_11289	31209	scaffold_1:2129994-2130959
			Mpu48918	estExt_fgenesh1_pg.C_150036	48918	scaffold_15:125924-129232
Oryza sativa ssp. *japonica*	Phytozome	8	Os01g25450		Os01g25450	Os01g25450
			Os02g35130		Os02g35130	Os02g35130
			Os03g13730		Os03g13730	Os03g13730
			Os03g61890		Os03g61890	Os03g61890
			Os04g36030		Os04g36030	Os04g36030
			Os05g05950		Os05g05950	Os05g05950
			Os10g40110		Os10g40110	Os10g40110
			Os12g09570		Os12g09570	Os12g09570
Ostreococcus tauri	Phytozome	2	Ota31832	300010386	31832	Chr_03.0001:627734-628921

			Ota359	gw1.15.00.5.1	359	Chr_15.0001:464848-466785
Physcomitrella patens	JGI	7	Ppa110926	e_gw1.1.373.1	110926	scaffold_1:2163123-2165948
			Ppa125298	e_gw1.54.185.1	125298	scaffold_54:65633-67998
			Ppa188734	estExt_gwp_gw1.C_1230061	188734	scaffold_123:544312-549289
			Ppa189669	estExt_gwp_gw1.C_1360053	189669	scaffold_136:412344-416976
			Ppa211678	estExt_Genewise1.C_720121	211678	scaffold_72:925178-928164
			Ppa216050	estExt_Genewise1.C_1230058	216050	scaffold_123:536812-542765
			Ppa216964	estExt_Genewise1.C_1360048	216964	scaffold_136:405460-410460
Populus trichocarpa	Phytozome	12	Ptr200841	gw1.IX.1306.1	200841	LG_IX:2278167-2280416
			Ptr200844	gw1.IX.1309.1	200844	LG_IX:2288274-2290556
			Ptr229304	gw1.X.4001.1	229304	LG_X:2214959-2217496
			Ptr249502	gw1.XIX.1902.1	249502	LG_XIX:8877842-8880823
			Ptr278713	gw1.210.26.1	278713	scaffold_210:68799-71192
			Ptr278721	gw1.210.34.1	278721	scaffold_210:76990-79395
			Ptr287977	gw1.41.462.1	287977	scaffold_41:1656754-1659198
			Ptr578875	eugene3.00181099	578875	LG_XVIII:12016135-12018384
			Ptr713242	estExt_Genewise1_v1.C_LG_IV1051	713242	LG_IV:15563804-15566490
			Ptr811548	fgenesh4_pm.C_scaffold_152000031	811548	scaffold_152:550193-553423
			Ptr816660	estExt_fgenesh4_pg.C_LG_II1667	816660	LG_II:14393384-14397862
			Ptr834816	estExt_fgenesh4_pm.C_LG_XIV0229	834816	LG_XIV:4474346-4479286
Selaginella moellendorffii	Phytozome	4	Smo160107	estExt_Genewise1.C_960141	160107	scaffold_96:616152-617676
			Smo170624	estExt_Genewise1Plus.C_120265	170624	scaffold_12:799985-802546
			Smo24614	gw1.72.111.1	24614	scaffold_72:898364-899172
			Smo446135	estExt_fgenesh2_pg.C_650095	446135	scaffold_65:780848-782802
Sorghum bicolor	Phytozome	7	Sb01g002230	Sb01g002230	5047777	chr_1:1861768-1866624
			Sb01g029790	Sb01g029790	5049109	chr_1:51888647-51892100
			Sb04g022760	Sb04g022760	5055262	chr_4:52343012-52348130
			Sb06g017450	Sb06g017450	5041878	chr_6:46721366-46722323
			Sb08g006190	Sb08g006190	5059442	chr_8:9176841-9180587
			Sb09g004020	Sb09g004020	5060122	chr_9:4478613-4483079

			Sb131445	fgenesh1_kg.C_chr_1000488	131445	chr_1:64780026-64784663
Tetrahymena thermophila	EnsemblProtists	1	Tth3818		3818.m00696	
Trichomonas vaginalis	NCBI	2	Tva85064		85064.m00040	
			Tva87871		87871.m00099	
Vitis vinifera		6	Vvi03602		GSVIVP00003602001	VVUNRG1506
	Phytozome		Vvi06412		GSVIVP00006412001	VV11RG0026
			Vvi14090		GSVIVP00014090001	VV16G0244
			Vvi21940		GSVIVP00021940001	VV18G1791
			Vvi27973		GSVIVP00027973001	VV7G0371
			Vvi36085		GSVIVP00036085001	VV3G0172
**Other species**						
Brassica napus	NCBI	1	BnaAAQ17548		AAQ17548	
Brassica rapa	NCBI	1	BraABK78687		ABK78687	
Carica papaya	NCBI	3	Cpa33_189	evm.model.supercontig_33.189	33.189	
			Cpa471_6	evm.model.supercontig_471.6	471.6	
			Cpa9_215	evm.model.supercontig_9.215	9.215	
Elaeis guineensis	NCBI	1	EguACF06616		ACF06616	
Medicago truncatula	NCBI	1	MtrACJ85286		ACJ85286	
Nicotiana tabacum	NCBI	1	NtaAAD09518		AAD09518	
Orychophragmus violaceus	NCBI	1	OviAAM77647		AAM77647	
Picea sitchensis	NCBI	6	PsiABK23761		ABK23761	
			PsiABK26629		ABK26629	
			PsiABR17678		ABR17678	
			PsiABR17719		ABR17719	
			PsiABR17900		ABR17900	
			PsiACN40681		ACN40681	
Pisum sativum	NCBI	2	PsaCAA82196		CAA82196	
			PsaCAA83453		CAA83453	
Ricinus communis	NCBI	6	RcoEEF30522	XP002531885	EEF30522	

			RcoEEF31623	XP002530763	EEF31623
			RcoEEF34118	XP002528280	EEF34118
			RcoEEF45536	XP002516922	EEF45536
			RcoEEF47115	XP002515131	EEF47115
			RcoEEF52958	XP002510771	EEF52958
Solanum tuberosum	NCBI	2	StuABA40447		ABA40447
			StuABB16976		ABB16976
Zea mays	NCBI	5	ZmaNP001105129		NP_001105129
			ZmaNP001105522		NP_001105522
			ZmaNP001131755		NP_001131755
			ZmaNP001147969		NP_001147969
			ZmaNP001148533		NP_001148533
